# No Evidence that Knops Blood Group Polymorphisms Affect Complement Receptor 1 Clustering on Erythrocytes

**DOI:** 10.1038/s41598-017-17664-9

**Published:** 2017-12-19

**Authors:** O. V. Swann, E. M. Harrison, D. H. Opi, E. Nyatichi, A. Macharia, S. Uyoga, T. N. Williams, J. A. Rowe

**Affiliations:** 1 Centre for Immunity, Infection and Evolution, Institute of Immunology and Infection Research, School of Biological Sciences, University of Edinburgh, Edinburgh, UK; 2 Clinical Surgery, University of Edinburgh, Edinburgh, UK; 3 Wellcome Trust Research Laboratories/Kenya Medical Research Institute, Centre for Geographic Medicine Research, Kilifi, Kenya; 4 Department of Medicine, Imperial College, London, UK; 5 Present Address: Burnet Institute for Medical Research and Public Health, Melbourne, Victoria, 3004 Australia

## Abstract

Clustering of Complement Receptor 1 (CR1) in the erythrocyte membrane is important for immune-complex transfer and clearance. CR1 contains the Knops blood group antigens, including the antithetical pairs Swain-Langley 1 and 2 (Sl1 and Sl2) and McCoy a and b (McC^a^ and McC^b^), whose functional effects are unknown. We tested the hypothesis that the *Sl* and *McC* polymorphisms might influence CR1 clustering on erythrocyte membranes. Blood samples from 125 healthy Kenyan children were analysed by immunofluorescence and confocal microscopy to determine CR1 cluster number and volume. In agreement with previous reports, CR1 cluster number and volume were positively associated with CR1 copy number (mean number of CR1 molecules per erythrocyte). Individuals with the *McC* ^*b*^ */McC* ^*b*^ genotype had more clusters per cell than *McC* ^*a*^/*McC* ^*a*^ individuals. However, this association was lost when the strong effect of CR1 copy number was included in the model. No association was observed between *Sl* genotype, sickle cell genotype, α+thalassaemia genotype, gender or age and CR1 cluster number or volume. Therefore, after correction for CR1 copy number, the *Sl* and *McCoy* polymorphisms did not influence erythrocyte CR1 clustering, and the effects of the Knops polymorphisms on CR1 function remains unknown.

## Introduction

**Figure 1:**
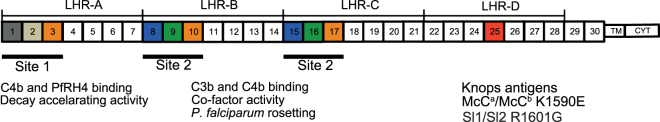
Diagram of the most common CR1 size variant (CR1*1). Adapted from Schmidt *et al*.^70^. The extracellular domain of CR1 is composed of 30 Complement Control Protein (CCP) domains organized into four “Long Homologous Repeats” (LHR). Two major functional sites are found within the CR1 molecule. Site 1 is located in LHR-A (CCP 1–3) and is the binding site for C4b and the *Plasmodium falciparum* invasion ligand PfRh4, and has decay accelerating activity. Site 2 is located in LHR-B (CCP 8–10) and LHR-C (CCP 15–17), and binds C3b and C4b, and interacts with *P. falciparum* infected erythrocytes to form rosettes. In addition, site 2 has Factor I-cofactor activity. SNPs determining the *Sl* and *McC* polymorphisms are localized in LHR-D (CCP 25, shown in red). CCP regions within Sites 1 and 2 that share high sequence identity (between 1 to 3 amino acid changes) are represented with identical shading. TM, transmembrane region; CYT, cytoplasmic tail.

Complement receptor 1 (CR1) is a polymorphic transmembrane glycoprotein expressed in humans on erythrocytes, most leucocytes and glomerular podocytes^1^. It is encoded by the *CR1* gene at the 1q32 locus, spanning approximately 133 kb and consisting of 39 exons^2^. Four size polymorphisms of CR1 have been identified, the most common form being CR1*1^3^. This classical form has a molecular weight of 190 kDa under non-reducing conditions, with an ectodomain comprising 30 short consensus repeats (SCRs), the first 28 of which are arranged into four long homologous repeats (LHRs) A-D (Fig. 1)^4^. Insertion or deletion of LHRs accounts for the variation seen in the molecule size^4^. Variation also occurs in erythrocyte CR1 copy number, with the mean number of CR1 molecules per cell varying between individuals from 50 to 1200^5^. A *HindIII* restriction fragment length polymorphism in intron 27 of the CR1 gene correlates with high (H allele) or low (L allele) CR1 expression on erythrocytes^6^. However, this polymorphism is not associated with CR1 copy number in African populations^7,8^.

CR1 is a key regulator of complement cascade activation, namely through accelerating the decay of C3 and C5 convertases thus reducing production of anaphylotoxins C3a and C5a^9^, and acting as a cofactor with Factor I to inactivate C3b and C4b to iC3b and iC4b^10^. These functions map to specific sites within the CR1 molecule, with site 1 (SCRs 1–3 in LHR-A) displaying high decay accelerating activity^11^ and binding C4b^12,13^, and site 2 (SCRs 8–10 in LHR B and SCRs 15–17 in LHR C) being responsible for cofactor activity with Factor I and the binding of C3b and C4b (Fig. 1)^12,13^.

The function of the LHR-D region of CR1 is unclear, but may include binding sites for C1q^14^ and Mannose Binding Lectin (MBL)^15^. SCR 25 in LHR-D also contains the Knops blood group antigens, which include the Swain-Langley (antithetical pairs Sl1 and Sl2) and McCoy (McC^a^ and McC^b^) antigens (Fig. 1)^16^. The *Sl2* allele results from a single nucleotide polymorphism (SNP) (rs17047661), encoding an amino acid change from arginine to glycine (R1601G), while the *McC* ^*b*^ allele results from a SNP 33 base pairs away (rs17047660), encoding an amino acid change from lysine to glutamic acid (K1590E) (Fig. 1)^16^. The functional effects of the *Sl2* and *McC* ^*b*^ polymorphisms remain unknown. Recent work investigating short recombinant proteins bearing the Sl and McC antigens found no difference between the variant forms in their affinities for immobilised C3b, C4b or C1q, nor in their co-factor activity for cleavage of C3b and C4b in conjunction with Factor I^17^.

A marked geographical difference is seen in the frequency of *Sl2* and *McC* ^*b*^ alleles, with the alleles being common in African populations and rare in Caucasians^18^. This has led to the suggestion that these polymorphisms arose due to selection pressure from malaria^19^, however, studies to date have been conflicting. An observational study from Western Kenya reported an association between the *Sl2/Sl2* genotype and a reduced odds ratio of cerebral malaria (OR 0.17, p = 0.02) when compared to the *Sl1/Sl1* genotype^20^. Recently, analysis of a large case-control study from Kilifi (eastern Kenya) identified an association between the *Sl2/Sl2* genotype and protection against cerebral malaria and death from malaria, but conversely found addition of the *McC* ^*b*^ allele to be associated with increased odds of cerebral malaria (Opi, Swann *et al*., submitted**)**. However, studies from The Gambia and Ghana did not find significant associations between the polymorphisms and severe malaria^21,22^.

In addition to complement regulation, CR1 on erythrocytes is responsible for immune adherence, the binding and transfer of opsonised immune complexes (ICs) from the circulation to resident macrophages in the liver and spleen for phagocytosis^23,24^. The clustering of CR1 in the erythrocyte membrane is thought to play a key role in its immune adherence function. On the resting erythrocyte membrane, CR1 is widely dispersed in small clusters, which coalesce into larger clusters when cross-linked by ligands or antibodies^25–31^. CR1 has also been demonstrated to co-immunoprecipitate with Fas-associated binding protein 1 (FAP-1) in the cytoskeleton fraction, which acts as a scaffolding protein for cross-linked CR1 and cluster formation^28^. It has been hypothesized that CR1 clustering after cross-linking by ligands could prevent ingestion of the erythrocyte during IC transfer to macrophages by concentrating opsonic stimuli in a few areas of the membrane^28^. Ligation of CR1 has also been demonstrated to increase erythrocyte deformability, which could allow IC-laden erythrocytes to traverse the hepatic and splenic capillaries and transfer their cargo to the resident macrophages more easily^29^.

The number of CR1 clusters per erythrocyte increases with CR1 copy number^25,32,33^, although CR1 copy number alone does not completely explain inter-individual variation in clustering, implying involvement of other factors^32^. The possibility that the *Sl2* polymorphism might affect CR1 clustering has been raised^34^, but never investigated. If the Knops blood group is indeed associated with malaria, there are a number of mechanistic possibilities that could include a role for variation in CR1 clustering. If the *Sl2* or *McCb* mutations influenced the size or number of CR1 clusters formed, this might influence the process of invasion of *Plasmodium falciparum* into the erythrocyte via CR1 “capping” of the merozoite^35^. Alternatively, CR1 clustering could be important in the pathogenesis of cerebral malaria. Children with cerebral malaria may have higher circulating levels of immune complexes (ICs) than children with other malaria disease syndromes^36^. ICs deposited on the endothelial surface can activate complement and stimulate the release of local pro-inflammatory mediators^37^. ICs can also cross-link Fc-receptors on monocyte and macrophages, stimulating production of tumour necrosis factor^38^, a cytokine whose relationship with cerebral malaria has been previously reported^39^. As avidity of IC binding to erythrocytes depends on CR1 clustering, clearance of ICs could potentially vary with different CR1 clustering patterns.

We therefore set up a study using blood samples from healthy children in Kilifi, Kenya to examine the hypothesis that the *Sl2* and *McC* ^*b*^ polymorphisms might influence erythrocyte CR1 clustering.

## Results

Erythrocyte CR1 clustering was assessed in blood samples from 125 Kenyan children with the homozygous Knops genotypes *Sl1/Sl1 McC* ^*a*^ */McC* ^*a*^, *Sl2*/*Sl2 McC* ^*a*^ */McC* ^*a*^ or *Sl2/Sl2 McC* ^*b*^ */McC* ^*b*^. These genotypes are hereafter referred to as *1a, 2a* and *2b* respectively. Only homozygous genotypes were studied in order to examine the associations of the “pure forms” of the polymorphisms on CR1 clustering. The characteristics of the children are summarised in Table 1; no significant differences were seen between the *1a, 2a* or *2b* genotype groups in terms of gender, age, ABO blood group, α^+^thalassaemia genotype or sickle cell genotype.

**Table 1:** Characteristics of individuals studied.

Characteristic	Total (n)	Genotype^#^			p value
		*1a* (n = 29) (%)	*2a* (n = 88) (%)	*2b* (n = 8) (%)	
Gender					
Male	73	17 (59)	51 (58)	5 (62.5)	1*
Female	52	12 (41)	37 (42)	3 (37.5)	
Age (months)					
Mean (SD)	NA	130 (34)	119 (32)	138 (36)	0.124^§^
ABO blood group					
O	68	16 (55)	47 (53)	5 (62.5)	0.930*
A	29	8 (28)	19 (22)	2 (25)	
B	23	5 (17)	17 (19)	1 (12.5)	
AB	5	0 (0)	5 (6)	0 (0)	
α+thalassaemia genotype‡					
αα/αα	49	12 (41)	33 (38)	4 (50)	0.857*
−α/αα	51	13 (45)	35 (40)	3 (37.5)	
−α/−α	25	4 (14)	20 (22)	1 (12.5)	
Sickle cell (HbS) genotype^‡^					
AA	108	25 (86)	77 (88)	6 (75)	0.547*
AS	17	4 (14)	11 (12)	2 (25)	
CR1 copy number					
Mean (SD)	NA	495 (204)	478 (221)	658 (305)	0.098^§^

^#^1a = *Sl1/Sl1, McC* ^*a*^/*McC* ^*a*^ genotype, 2a = *Sl2/Sl2, McC* ^*a*^/*McC* ^*a*^ genotype, 2b = *Sl2/Sl2, McC* ^*b*^/*McC* ^*b*^ genotype.

*Fisher’s exact test, ^§^One-way ANOVA.

^‡^α^+^thalassaemia and sickle cell genotype were included in the study as both have previously been reported to influence CR1 copy number^40,42,63^.

SD, standard deviation. NA, not applicable.

**Figure 2:**
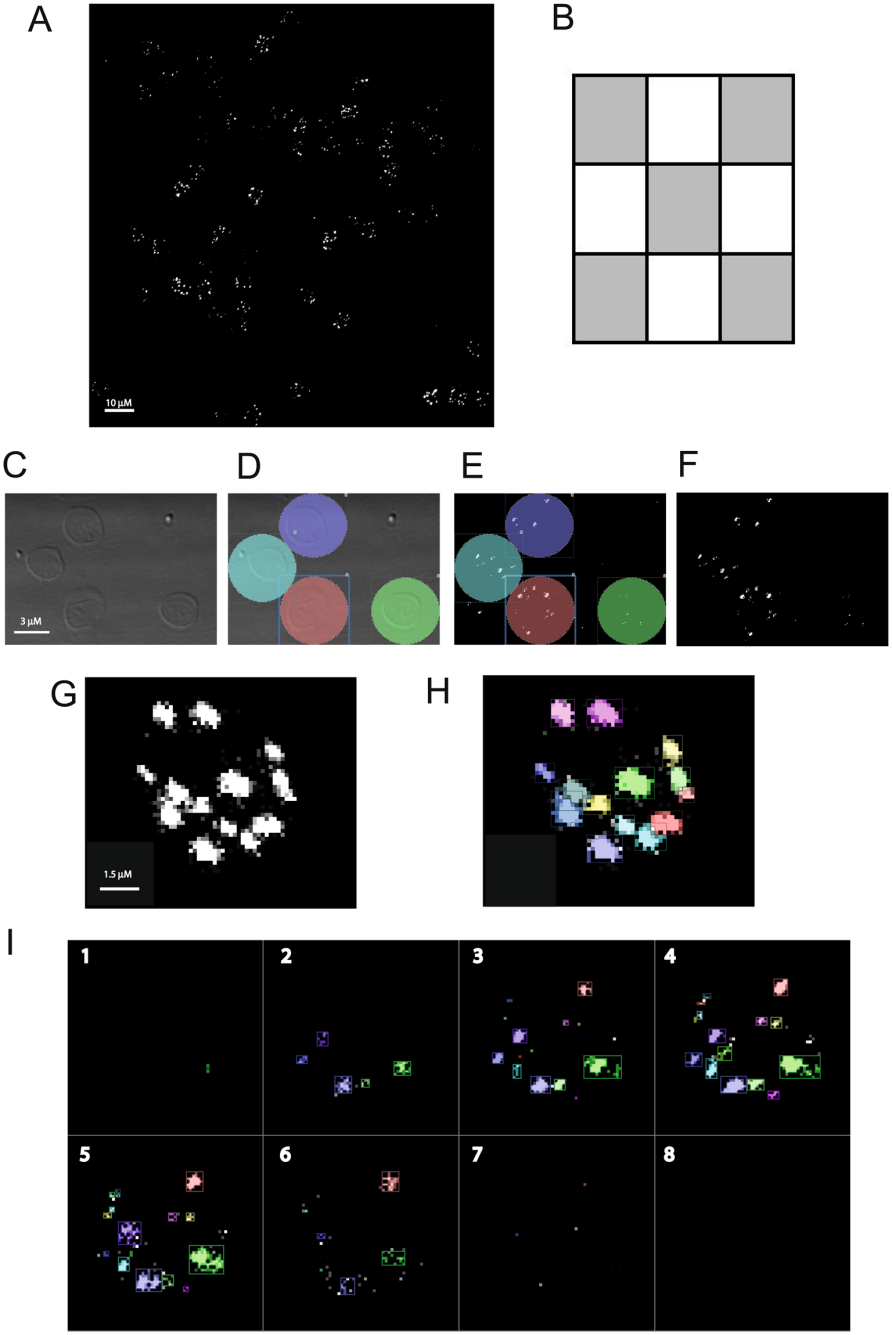
Immunofluorescent staining of erythrocyte CR1 clusters and image analysis. (**A**) Erythrocyte CR1 clusters were visualised by staining with 5 μg/ml of CR1 monoclonal antibody J3D3, followed by 4 μg/ml of Alexa Fluor 488 goat anti-mouse IgG. See methods for full details. (**B**) For each donor, five confocal microscopy images were taken from the central area of the microscope slide as indicated by the grey shaded areas. Each image was a Z-stack comprising 8–10 steps. (**C–F)** To illustrate the image analysis process, a section containing 4 erythrocytes from one of the 5 images from a single donor is shown. Overall, at least 200 erythrocytes were examined per donor. (**C**) For each image, the bright-field view was used to identify the positions of erythrocytes. (**D**) Non-touching cells were then stamped. (**E)** The stamped region was then applied to the fluorescent image. (**F**) Cluster numbers and volumes within the stamped regions were assessed by an automated protocol using Volocity software as described in the methods. (**G)** An example of the clusters identified in a single erythrocyte. (**H)** The same erythrocyte as shown in G, with distinct clusters being shown in different colours. (**I**) A different erythrocyte with each of the 8 steps that comprise the Z-stack shown separately (1 to 8). These images can also be viewed as a video (Supplementary Videos 1 and 2).

In order to examine CR1 clusters, we carried out immunofluorescent staining of unfixed erythrocytes with a monoclonal antibody to CR1 (Fig. 2A), and analysed confocal microscopy images with an automated protocol developed using Volocity Image Analysis software (version 6.3, Perkin Elmer) to determine CR1 erythrocyte cluster number and volume (Fig. 2B–H and Supplementary Videos 1 and 2).

### Increased CR1 copy number in individuals with the *McCb/McCb* genotype

**Figure 3:**
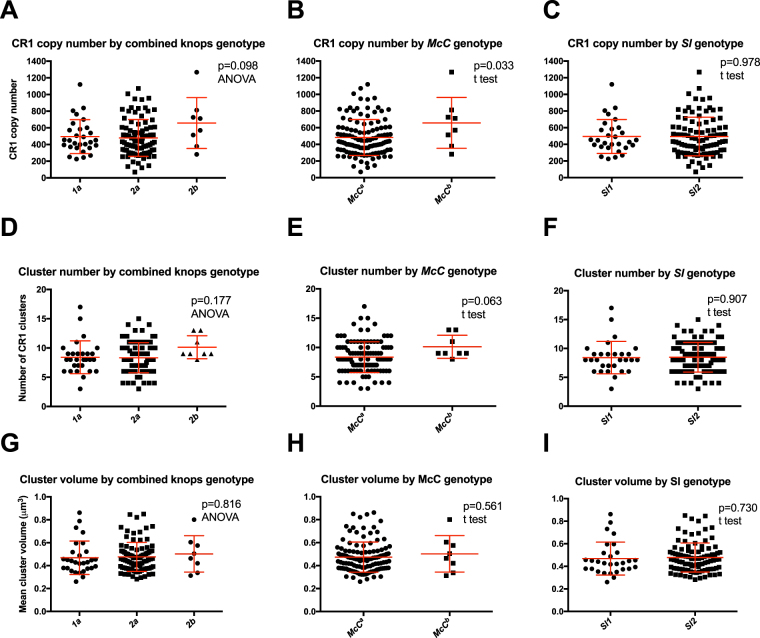
The *McC* ^*b*^ genotype is associated with increased CR1 copy number and cluster number. Preliminary exploration of the association of Swain Langley (*Sl*) and McCoy (*McC*) genotypes with CR1 copy number (mean number of CR1 molecules per erythrocyte), median CR1 cluster number per cell and mean cluster volume. Panels (**A–C**) Relationship between CR1 copy number and (**A**) combined *Sl* and *McC* genotype (*1a* = *Sl1/Sl1 McC* ^*a*^ */McC* ^*a*^, *2a* = *Sl2/Sl2 McC* ^*a*^ */McC* ^*a*^ and *2b* = *Sl2/Sl2 McC* ^*b*^ */McC* ^*b*^), (**B**) *McC* genotype and (**C**) *Sl* genotype. Panels (**D–F**) Relationship between median number of CR1 clusters per cell and (**D**) combined *Sl* and *McC* genotype, (**E**) *McC* genoype and (**F**) *Sl* genotype. Panels (**G–I**) Relationship between mean CR1 cluster volume per cell and (**G**) combined *Sl* and *McC* genotype, (**H**) *McC* genoype and (**I**) *Sl* genotype. Red bars indicate mean and standard deviation, with statistical testing by t test or ANOVA as indicated. Individuals with the *McC* ^*b*^ genotype had significantly higher CR1 copy numbers than *McC* ^*a*^ individuals, and showed a trend towards higher CR1 cluster numbers, although this was not statistically significant.

CR1 copy number (mean number of CR1 molecules per erythrocyte) positively correlates with number of CR1 clusters^25,32,33^ and was therefore measured in all individuals. We first examined the association of CR1 copy number and donor variables in univariate analyses. We observed that individuals with the *2b* genotype showed a trend towards  higher CR1 copy number than individuals with *1a* and *2a* genotypes (Table 1 and Fig. 3A, p = 0.098 by ANOVA). This was driven by the *McC* component of the genotype, as individuals with the *McC* ^*b*^ */McC* ^*b*^ genotype had a significantly higher CR1 copy number than *McC* ^*a*^ */McC* ^*a*^ (mean 658 copies per cell versus 483 copies per cell, p = 0.033, t-test, Fig. 3B). The *Sl* component of the genotype was not significantly associated with CR1 copy number (p = 0.978, t-test, Fig. 3C). Age, gender, ABO blood group and sickle cell trait showed no significant association with CR1 copy number in this study. Individuals who were homozygotes for *α* ^+^thalassaemia had a lower CR1 copy number (441) than those who were heterozygotes (495) or who had normal α globin (520), however this was not significant (p = 0.366, ANOVA, Figure S1).

### Increased CR1 cluster number in individuals with the *McCb/McCb* genotype

As previously reported^26,29,32^, the number of CR1 clusters per cell varied widely within each individual. Overall, the number of clusters per cell ranged from 0 to 43, with the maximum number of clusters per cell ranging from 15 to 43 amongst different individuals (Figure S2). The volume of CR1 clusters also varied within individuals, with the largest clusters being > 3μm^3^ (Figure S3).

Preliminary analysis of the relationship between Knops genotype and erythrocyte CR1 cluster number was conducted by ANOVA, with data summarised at the level of the individual donor (i.e. using a single data point per donor, representing the median cluster number for that donor). These data were suggestive of a higher cluster number in *2b* individuals, driven by the *McC* ^*b*^ genotype, although this was not statistically significant (Fig. 3D–F). Similar analysis by ANOVA of the relationship between Knops genotype and mean CR1 cluster volume revealed no significant associations (Fig. 3G–I). There were also no significant associations between CR1 cluster number (Figure S4) or cluster volume (Figure S5) and age, gender, ABO blood group, sickle cell genotype and *α* ^+^thalassaemia genotype.

**Figure 4:**
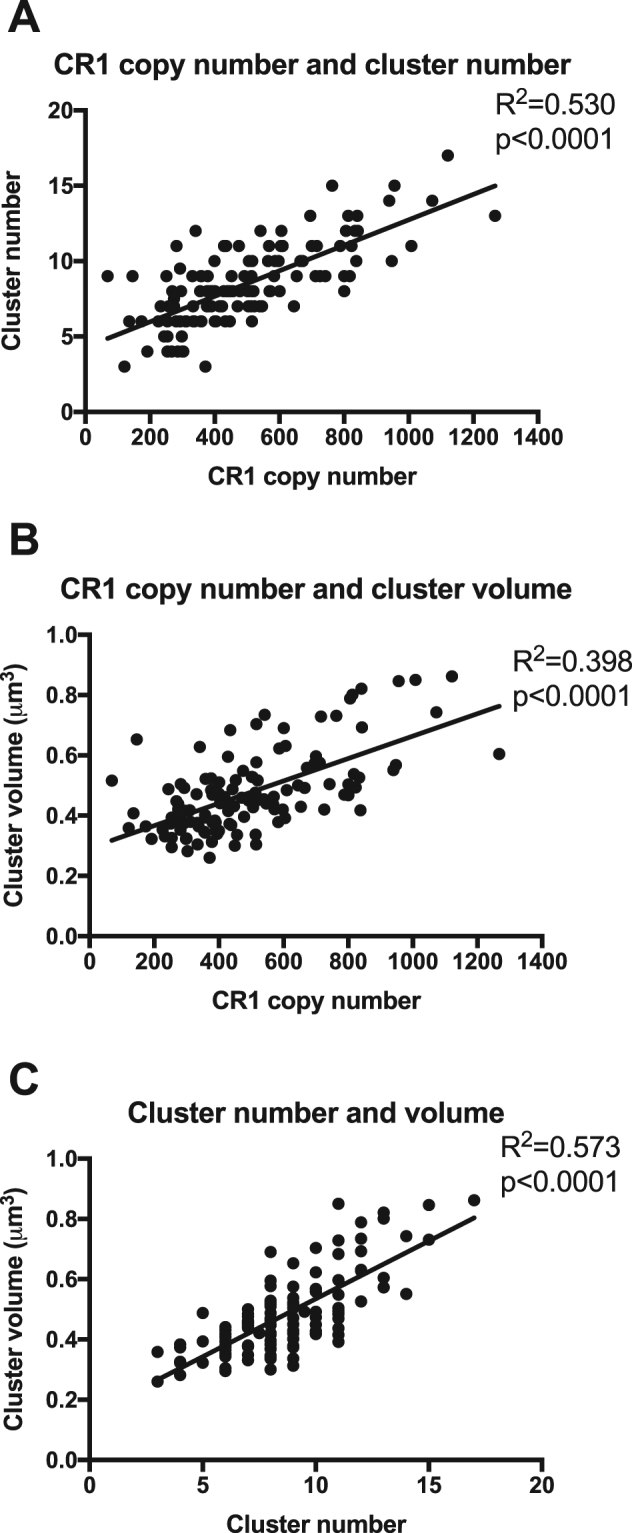
Positive correlation between CR1 copy number, cluster number and cluster volume. (**A**) Scatter plot of median CR1 cluster number per cell by CR1 copy number (mean number of CR1 molecules per cell) with linear regression. Each dot represents an individual donor. (**B**) Scatter plot of mean CR1 cluster volume by CR1 copy number with linear regression. Each dot represents an individual donor. (**C**) Scatter plot of mean CR1 cluster volume by median CR1 cluster number with linear regression. Each dot represents an individual donor. There were significant positive correlations between all three variables studied.

In agreement with previous findings^32,33^, a strong positive linear correlation was observed between CR1 cluster number and CR1 copy number (R^2^ = 0.530, p < 0.001, Fig. 4A), and a positive correlation was also observed between CR1 cluster volume and CR1 copy number (R^2^ = 0.398, p < 0.001, Fig. 4B). Cluster number and cluster volume were also strongly positively correlated (R^2^ = 0.573, p < 0.001, Fig. 4C). Because of the potential confounding effect of CR1 copy number on the relationship between Knops genotype and CR1 cluster number and volume, more detailed statistical analyses were carried out.

### No evidence of an association between Knops genotype and CR1 cluster number after adjustment for CR1 copy number

A hierarchical mixed effects Poisson regression analysis was constructed to include all available data at the level of the cell (using at least 200 data points per individual donor, representing the CR1 cluster number for each cell imaged) and to allow for adjustment for CR1 copy number. The analysis examined each donor variable (combined *Sl* and *McC* genotype, *Sl* genotype alone, *McC* genotype alone, gender, age, ABO blood group, α^+^thalassaemia genotype or sickle cell genotype) in turn. Each donor variable was specified as a fixed effect and donor identifier and date of assay were specified as random effects. The relationship between each donor variable and the number of CR1 clusters per cell was reviewed both before and after adjustment for CR1 copy number.

Prior to inclusion of CR1 copy number in the model, the *2b* genotype was associated with a higher number of clusters per cell than the *1a* genotype (multiplicative increase of 1.200 (95% CI 0.997–1.443, p = 0.053, Table 2)). When broken down by component genotypes, this association was driven by the *McC* component rather that the *Sl* component, with *the McC* ^*b*^ */McC* ^*b*^ genotype associated with a significantly higher number of clusters per cell than the *McC* ^*a*^ */McC* ^*a*^ genotype (multiplicative increase = 1.216, 95% CI 1.027–1.439, p = 0.023, Table 2). No significant associations were seen for gender, age, ABO blood group, α^+^thalassaemia genotype or sickle cell genotype.

**Table 2:** Effect of donor variables on cluster number from mixed effects Poisson regression. The rate ratio is the multiplicative change from the reference group.

Cluster number			Without adjustment for CR1 copy number			With adjustment for CR1 copy number		
Variable	Median	IQR	Rate Ratio	95% CI	p value	Rate Ratio	95% CI	p value
Combined *Sl/McC* genotype^#^								
*1a*	8	7–9	Reference	NA	NA	Reference	NA	NA
*2a*	8	7–10	0.982	0.888–1.085	0.717	0.992	0.920–1.069	0.831
*2b*	9	9–11.5	1.200	0.997–1.443	0.053	1.043	0.906–1.201	0.557
Swain-Langley component alone								
*Sl1/Sl1*	8	7–9	Reference	NA	NA	Reference	NA	NA
*Sl2/Sl2*	8	7–10	1.000	0.904–1.106	0.998	0.997	0.925–1.073	0.926
McCoy component alone								
*McC* ^*a*^ */McC* ^*a*^	8	7–10	Reference	NA	NA	Reference	NA	NA
*McC* ^*b*^ */McC* ^*b*^	9	9–10.5	**1.216**	**1.027–1.439**	**0.023**	1.049	0.921–1.195	0.470
Gender								
Female	8	7–10	Reference	NA	NA	Reference	NA	NA
Male	8	6–10	0.970	0.891–1.056	0.488	1.014	0.952–1.081	0.669
ABO blood group								
O	8	7–10.25	Reference	NA	NA	Reference	NA	NA
A	8	7–9	1.030	0.926–1.146	0.587	1.044	0.966–1.129	0.277
B	8	6–10.5	0.970	0.864–1.089	0.604	1.019	0.936–1.109	0.669
AB	8	8–10	0.979	0.784–1.222	0.851	1.053	0.895–1.241	0.532
α+thalassaemia genotype								
αα/αα	9	7–11	Reference	NA	NA	Reference	NA	NA
−α/αα	8	7–9	0.934	0.846–1.031	0.176	0.982	0.911–1.058	0.631
−α/−α	8	6–10	0.926	0.824–1.040	0.194	1.011	0.926–1.104	0.808
Sickle cell (HbS) genotype								
AA	8	7–10	Reference	NA	NA	Reference	NA	NA
AS	8	7–9	0.965	0.855–1.089	0.562	0.989	0.904–1.081	0.800
**Age (years)** Change in CR1 cluster number with each additional year of age			1.006	0.991–1.021	0.461	1.001	0.990–1.013	0.822
**CR1 copy number** Change in CR1 cluster number with each additional 100 CR1 copies per cell			NA	NA	NA	**1.084**	**1.067–1.101**	**<0.001**

^**#**^1a = *Sl1/Sl1*, *McC* ^*a*^ */McC* ^*a*^ genotype, 2a = *Sl2/Sl2*, *McC* ^*a*^ */McC* ^*a*^ genotype, 2b = *Sl2/Sl2*, *McC* ^*b*^ */McC* ^*b*^. IQR = inter-quartile range, CI = confidence interval by Wald. NA, not applicable.

The correlation between erythrocyte CR1 copy number and number of clusters per cell was strong, with each increase in CR1 copy number of 100 being associated with a multiplicative increase in CR1 cluster number of 1.084 (95% CI 1.067−1.101, p < 0.001). Adjustment for CR1 copy number obscured any association between cluster number and *McC* genotype (Table 2). This suggests that the original association was likely to be driven by a difference in CR1 copy number, rather than any unique effect of the *McC* genotype itself.

### No evidence of an association between Knops genotype and erythrocyte CR1 cluster volume after adjustment for CR1 copy number

Similarly, a more detailed hierarchical mixed effects linear regression analysis was constructed to include all available data at the level of the individual clusters, and to allow for adjustment for CR1 copy number. Donor variables (combined *Sl* and *McC* genotype, *Sl* genotype alone, *McC* genotype alone, gender, age, ABO blood group, α^+^thalassaemia genotype or sickle cell genotype) were examined individually as fixed effects, while cell identifier and donor identifier were specified as random effects (Table 3). The relationship between each donor variable and CR1 cluster volume was reviewed both before and after adjustment for CR1 copy number.

**Table 3:** Effect of donor variables on cluster volume from mixed effects linear regression. The coefficient is the absolute change from the reference group.

Variable	Mean cluster volume (µm^3^)	SD	Without adjustment for CR1 copy number			With adjustment for CR1 copy number		
			Coefficient	Standard error	t value	Coefficient	Standard error	t value
Combined genotype^#^								
*1a*	0.468	0.146	Reference	NA	NA	Reference	NA	NA
*2a*	0.476	0.127	0.012	0.029	0.400	0.016	0.022	0.717
*2b*	0.502	0.160	0.035	0.054	0.658	−0.029	0.042	−0.708
Sl component alone								
*Sl1/Sl1*	0.468	0.146	Reference	NA	NA	Reference	NA	NA
*Sl2/Sl2*	0.478	0.130	0.014	0.029	0.475	0.012	0.022	0.552
McC component alone								
*McC* ^*a*^ */McC* ^*a*^	0.474	0.131	Reference	NA	NA	Reference	NA	NA
*McC* ^*b*^ */McC* ^*b*^	0.502	0.160	0.027	0.049	0.545	−0.041	0.038	−1.08
Gender								
Female	0.486	0.126	Reference	NA	NA	Reference	NA	NA
Male	0.468	0.137	−0.022	0.024	−0.895	−0.011	0.019	−0.584
ABO blood group								
O	0.469	0.137	Reference	NA	NA	Reference	NA	NA
A	0.476	0.100	0.008	0.030	0.264	0.029	0.023	1.275
B	0.502	0.164	0.032	0.033	0.994	**0.057**	**0.025**	**2.305**
AB	0.451	0.083	−0.022	0.063	−0.355	0.012	0.047	0.262
α+thalassaemia genotype								
αα/αα	0.493	0.139	Reference	NA	NA	Reference	NA	NA
−α/αα	0.475	0.136	−0.022	0.027	−0.805	−0.008	0.021	−0.401
−α/−α	0.444	0.109	−0.051	0.033	−1.54	−0.017	0.026	−0.654
Sickle cell (HbS) genotype								
AA	0.472	0.133	Reference	NA	NA	Reference	NA	NA
AS	0.500	0.133	0.031	0.035	0.880	0.029	0.027	1.089
**Age (years)** Change in cluster volume with each additional year of age			−0.002	0.004	−0.560	−0.005	0.003	−1.411
**CR1 copy number** Change in cluster volume with each additional 100 CR1 copies per cell			NA	NA	NA	**0.039**	**0.004**	**9.446**

^**#**^ *1a* = *Sl1/Sl1*, *McC* ^*a*^ */McC* ^*a*^ genotype, *2a* = *Sl2/Sl2*, *McC* ^*a*^ */McC* ^*a*^ genotype*, 2b* = *Sl2/Sl2*, *McC* ^*b*^ */McC* ^*b*^. SD, standard deviation. NA, not applicable.

No donor variables were found to have a significant association with cluster volume before adjustment for CR1 copy number (i.e. t values were all <1.96 or >−1.96). The correlation between CR1 copy number and cluster volume was extremely strong, with each increase in CR1 copy number of 100 being associated with an absolute increase in CR1 cluster volume of 0.039 µm^3^ (t value 9.446, Table 3. Bootstrapped p value <0.001). Adjustment for CR1 copy number had no impact on the non-significant relationships between cluster volume and combined *Sl* and *McC* genotype, *Sl* genotype alone, *McC* genotype alone, gender, age, α^+^thalassaemia genotype or sickle cell genotype (Table 3). However, in models adjusting for CR1 copy number, the B blood group was associated with an absolute increase in cluster volume of 0.057 µm^3^ when compared to the reference O blood group (t value = 2.305, Table 3. Bootstrapped p value = 0.028).

## Discussion

In agreement with existing literature, we report that both the number of CR1 clusters per erythrocyte and CR1 cluster volume are highly correlated with an individual’s erythrocyte CR1 copy number^25,32,33^. The strength of association between CR1 copy number and cluster number was such that the inclusion of CR1 copy number in the statistical model obscured any other associations. However, on review of individual explanatory variables (without CR1 copy number in the model), the *McC* ^*b*^ */McC* ^*b*^ genotype was associated with significantly higher cluster number than the *McC* ^*a*^ */McC* ^*a*^ genotype. Individuals of the *McC* ^*b*^ */McC* ^*b*^ genotype were found to have a significantly higher CR1 copy number than *McC* ^*a*^ */McC* ^*a*^, which is in agreement with a larger analysis of Kenyan children^40^. As such, it is likely that the association of the *McC* ^*b*^ */McC* ^*b*^ genotype with increased CR1 cluster number was due to the higher CR1 copy numbers in *McC* ^*b*^ */McC* ^*b*^ individuals, rather than being a distinct biological effect of the variant itself. Exactly how the *McC* ^*b*^ polymorphism influences erythrocyte CR1 copy number is unknown, but could be due to resistance to cleavage by tryptic proteases resulting from the lysine to glutamic acid (K1590E) mutation, as discussed previously^40^.

In contrast to existing literature^40,41^, we found no evidence of an association between donor age and CR1 copy number. However, the previous studies included children between birth and 13 years^40^ or birth and 32 years^41^, with the major changes occurring during the first four years of life. The narrower age range of our study (5.7 years –12.7 years) may explain why no association between age and CR1 copy number was detected. We also found no significant effect of *α* ^+^thalassaemia genotype on CR1 copy number, unlike other studies which showed lower CR1 copy number in *α* ^+^thalassaemia homozygotes compared to normal individuals in Papua New Guinea^42^ and Kenya^40^. Whilst we also observed a lower CR1 copy number in *α* ^+^thalassaemia homozygote individuals, the small sample size in our study may have prevented this from reaching statistical significance.

One unexpected finding from this study was that blood group B was associated with significantly larger CR1 clusters than the reference blood group O. There is no known link between ABO blood group and CR1 function, and further work will be required to determine if this is a reproducible finding, and if so, to determine its biological significance.

Owing to the high level of within and between donor variation, this was a challenging assay to develop and interpret. The sample size of this study was small, in particular for the 2b genotype, which is a limitation of this study. However, given the logistics of sampling (see methods), it was not possible to recruit more individuals prospectively. Another consideration is that although all children positive for malaria on a thick film were excluded, information on other infective or inflammatory conditions that might influence CR1 copy number was not available. Therefore, it is possible that the CR1 copy number of some children may have been transiently altered by concurrent conditions. However, all children were sampled in the community and were not inpatients in health centres, therefore were in broadly good health.

The use of field samples also conferred other limitations on our study. When carrying out the confocal imaging in Edinburgh, we used conservative imaging settings to avoid photo-bleaching the slides during image acquisition. This was done in case repeated imaging was needed, as the slides themselves could not be replaced. Therefore, it is possible that we may have under-sampled the immunofluorescent slides, resulting in missing very small clusters. As temperature has been reported to affect cluster size in some studies^43^, the use of physiological temperature during the assay (rather than 4 °C as used here) may have increased the relevance of the findings, as may the use of a physiological ligand to induce clustering (e.g. C3b covered beads or immune complexes). Furthermore, the J3D3 monoclonal antibody used here recognises multiple epitopes in LHRs A, B and C^44^, and results could therefore be influenced by CR1 size polymorphisms (allotype), which was not accounted for here. An antibody with a single epitope on CR1 that would not be affected by CR1 allotype^43^ could be used in future studies. Other technical aspects of the assay may have affected the results shown here. For example, the mounting medium used after staining and preparing the thin blood films can cause lysis of the erythrocytes, potentially bringing the top and bottom membranes closer together. This could cause overlap of some clusters making them appear fewer or larger than *in vivo*. Despite these limitations, our methods were consistent throughout all samples, thus allowing for direct comparison between the different genotypes.

The functional effects of the *Sl* and *McC* polymorphisms remain elusive. Laboratory evidence suggests that *Sl2* may affect rosetting (the binding of *Plasmodium falciparum* infected erythrocytes to uninfected erythrocytes)^19^, a parasite virulence factor associated with severe malaria in African children^45^. Rosetting occurs when *P. falciparum* Erythrocyte Membrane Protein 1 (PfEMP1) expressed on the surface of infected erythrocytes binds to uninfected erythrocyte receptors, and is thought to involve site 2 on CR1 (the C3b binding site) in LHR-B and LHR-C^46^. Erythrocytes from *Sl2/Sl2* donors rosette less well than erythrocytes from *Sl1/Sl1* donors when PfEMP1 is expressed heterologously on the surface of COS-7 cells^19^. However, a study using short recombinant proteins of the *Sl* and *McC* variants found no difference in their abilities to disrupt rosettes^17^. Further investigation of the effects of the Knops polymorphisms on *P. falciparum* rosetting is required.

In addition to its role in rosetting, CR1 has also been identified as a sialic acid-independent invasion receptor for *P. falciparum* ^47^, via the reticulocyte-binding–like homolog 4 (PfRh4)^48^. The binding of PfRh4 has been mapped to site 1 in LHR-A^49–51^. Immuno-fluorescent staining of freshly released invasive merozoites showed CR1 on erythrocytes to be more intense around the merozoite, referred to by the authors as “capping,” suggesting CR1 clustering may have a role in invasion^47^. However, the previously cited study using short recombinant proteins of the *Sl* and *McC* variants also found no difference in their abilities to inhibit erythrocyte invasion by *P. falciparum* ^17^.

Interestingly, the binding site for mannose binding lectin (MBL) has been mapped to CCP 24–25, in close proximity to the *Sl* and *McC* polymorphisms^52^. MBL may influence malaria in Ghanaian children, with alleles responsible for low levels of MBL associated with increased parasite load, lower blood glucose levels and increased odds of severe malarial anaemia^53,54^. It has also been suggested that MBL may opsonize *P. falciparum* infected erythrocytes^53^ or merozoites^55^. However, whether the *Sl* or *McC* polymorphisms have functional effects on MBL interactions with CR1 has not yet been explored.

In summary, after accounting for the effect of CR1 copy number, there was no significant association between *Sl* or *McC* genotypes (separately or in combination) and erythrocyte CR1 cluster number or cluster volume in Kenyan children. The effects of the *Sl* and *McC* Knops blood group polymorphisms on CR1 function remain unknown.

## Materials and Methods

### Study Population and Ethical Approval

The Immunology Cohort Study Group in Kilifi, Kenya comprises 1041 children resident in the Junju or Ngerenya regions of the Kilifi Demographic Surveillance System (KDSS)^56,57^. The Kilifi district borders the Indian Ocean and the local economy depends on subsistence farming^57^. It is a malaria endemic area with seasonal transmission after the long (April -June) and short (October-November) rainy seasons, although recently malaria transmission has fallen in the area^58^. Ethical approval for this study was obtained from the KEMRI Ethical Review Committee and all methods were performed in accordance with the relevant guidelines and regulations. Following informed consent from a parent or guardian, blood samples were obtained by venepuncture between 2^nd^ and 30^th^ April 2013 (inclusive).

### Donor selection and genotyping

Donors had previously been genotyped for *Sl* and *McC* polymorphisms using the SEQUENOM iPLEX^®^ Gold platform^59^, for sickle cell (HbS) and α^+^thalassaemia genotype (–α3.7 deletion) by PCR^60,61^ and typed for ABO blood group by standard haemagglutination methods^62^. Our investigation was limited to the *Sl* and *McC* homozygote genotypes, *1a* (*Sl1/Sl1 McC* ^*a*^ */McC* ^*a*^), *2a* (*Sl2/Sl2 McC* ^*a*^ */McC* ^*a*^) and *2b* (*Sl2/Sl2 McC* ^*b*^ */McC* ^*b*^). The *1b* genotype does not occur because the *McC* ^*b*^ polymorphism is only found on the background of the *Sl2* variant^16,18^. 148 children in the cohort with *1a, 2a* or *2b* genotypes, and with complete data on α^+^thalassaemia and sickle cell status (both reported to influence CR1 copy number^40,42,63^) were eligible for inclusion. Nine children did not attend for venepuncture, 12 were excluded because they were positive for *P. falciparum* on thick blood smear, and two were accidently discarded, giving 125 samples for analysis (29 with genotype *1a*, 88 with genotype *2a* and eight with genotype *2b*).

### Sample collection and processing

4.4 mls of venous blood was collected into a lithium heparin tube, centrifuged at 400 g for 10 minutes and plasma removed. The sample was then made up to 5 mls with R2 medium (500 ml RPMI (catalogue number R0883, Sigma-Aldrich, Missouri, USA) with final concentration of 0.02 M Hepes (Gibco, New York, USA), 0.1 mM L-glutamine (Gibco, New York, USA), 1% vol/vol Penicillin–Streptomycin Solution Hybri-Max **(**Sigma-Aldrich, Missouri, USA) and 2% pooled human AB serum). The cell suspension was layered onto 3 ml of Lymphoprep (PROGEN Biotechnik, Heidelberg, Germany) and centrifuged at 1000 g for 20 minutes at 20 °C. The white cell layer was removed and the resulting erythrocytes washed twice in R2 medium without serum by centrifugation at 400 g for 5 minutes. They were then adjusted to 4% haematocrit by adding R2 medium without serum. The clustering assay was subsequently performed on these unfixed cells. As storage of blood samples can affect CR1 copy number and function^43,64,65^, all clustering assays were performed within eight hours of venepuncture.

### CR1 copy number determination

An aliquot of washed erythrocytes from above was fixed in 5% formaldehyde (Sigma-Aldrich, Missouri, USA) on the day of venepuncture and CR1 copy number was determined within 8 weeks as previously described in detail^65^. Briefly, this method uses flow cytometry to quantify CR1 copy number on fixed erythrocytes using a standard curve derived from donors with known CR1 copy numbers that have been fixed at the same time as the samples. Erythrocytes were stained with 0.5 μg/ml J3D3 anti-CR1 monoclonal antibody (GTX44217, GeneTex, California, USA), followed by 5 μg/ml of Alexa Fluor 488-conjugated goat anti-mouse IgG (A-11001, ThermoFisher Scientific, Paisley, UK) and the mean fluorescence intensity determined on a FC500 flow cytometer (Beckman-Coulter Inc.). Examples of the gating strategy and staining of low, intermediate and high CR1 standard donors are shown in Figure S6.

### Immunofluorescent staining of CR1 clusters

The IgG_1_ mouse anti-CR1 monoclonal antibody J3D3 was used to stain CR1 clusters, as it does not recognise epitopes in the LHR-D region^44^, so was considered unlikely to influence any effect the *Sl* or *McC* polymorphisms might have on clustering. Two aliquots from each sample of washed, unfixed erythrocytes at 4% haematocrit in PBS/1% BSA were incubated with 5 μg/ml of either J3D3 or IgG_1_ mouse anti-human isotype control (MCA928, AbD Serotec, Kidlington, UK) to give a final sample volume of 50 μl. Samples were incubated at 4 °C for one hour (resuspended every ten mins) and washed three times in 1 ml cold PBS (Oxoid, Basingstoke, UK). 50 μl of 4 μg/ml Alexa Fluor 488-conjugated goat anti-mouse secondary antibody was added and the sample was incubated at 4 °C in the dark for one hour (resuspended every ten mins) and subsequently washed three times in 1 ml cold PBS. The sample was resuspended at 30–40% haematocrit, smeared on a glass slide, air-dried, mounted under a 22 mm × 22 mm glass coverslip with 10 μl of DABCO-glycerol (2.5 mg DABCO (Sigma-Aldrich, Poole, UK), 0.5 ml glycerol and 0.5 ml PBS) and sealed with nail varnish. All steps to this point were undertaken at the KEMRI-Wellcome Trust Research Unit, Kilifi and the resultant slides were shipped to Edinburgh for confocal microscopy.

### Confocal microscopy

Slides were imaged with a Leica SP5 confocal microscope using the glycerol × 63 objective, argon laser and differential interference contrast settings (now referred to as “bright field”). Five images per slide (Fig. 2A shows a representative image) were taken from predefined areas (Fig. 2B). To prevent bias for stained cells, each slide was navigated using the bright field image, ensuring that only erythrocyte outlines, rather than CR1 clusters, were visible. To minimise photo-bleaching, a Z-stack of images was taken at 0.8 μm intervals and between eight to ten steps were acquired per stack (an example of the 8 steps comprising a Z-stack are shown in Fig. 2I and Supplementary Videos 1 and 2). The images in theZ-stack were then merged into one 3D image using Volocity 3D Image Analysis Software version 6.3 (PerkinElmer, Massachusetts, USA). Slides were imaged blind to donor information.

### Image Analysis

Using the bright field image (to obscure CR1 clusters and increase objectivity) (Fig. 2C), a region of interest stamp 9 µm in diameter (area 63.6 µm^2^, slightly larger than an erythrocyte) was used to identify individual erythrocytes that were not touching other cells (Fig. 2D). Approximately 40 erythrocytes per image were stamped. Image analysis was then carried out within the stamped areas in the fluorescent channel (Fig. 2E,F). An automated software protocol was developed using the “Find objects” task in the “Measurements” module in Volocity version 6.3 (Perkin Elmer), and was used to determine the number of CR1 clusters per erythrocyte (Fig. 2G,H), and to calculate the volume of each cluster. The protocol identified objects within the stamped area based on a minimum size threshold and subsequently separated touching objects using an object size guide. To establish the protocol settings, five cells from five separate donors (total cell number = 25) were reviewed. Settings over a range from 0.01 to 2.56 μm^3^ were examined for both minimum size and object size guide. A minimum size threshold of 0.06 μm^3^ and object size guide of 0.06 μm^3^ gave the optimal correlation with number of clusters identified by eye (Pearson’s correlation coefficient of 0.927 and r^2^ of 0.859), therefore these settings were used throughout. To account for background fluorescence, 30 maximum fluorescent intensity (MFI) readings were taken from areas of cells without visible CR1 clusters for each donor. The median of these readings was calculated for each individual to give the background MFI. The median of all donor medians was then calculated, giving a universal background correction factor (median = 554, IQR = 554–693) that was subtracted from all samples before running the Volocity protocol.

In order to determine the optimal number of cells to image from each donor, 400 erythrocytes were imaged from a single donor. The standard error of the mean (SEM) of the number of CR1 clusters per erythrocyte was plotted against the number of erythrocytes imaged (Figure S7). It was decided to image 200 erythrocytes for each donor, as gains in SEM beyond this point were small.

The reproducibility of the assay was assessed by imaging a blood sample taken from a single donor at the same time each day for three consecutive days. Neither the median number of CR1 clusters per cell, nor the mean CR1 cluster volume was significantly different across the three days (Kruskal-Wallis test p = 0.337 and 0.054 respectively) (Figure S8).

### Statistical analyses

Our experiments generated data on individual CR1 clusters from at least 200 erythrocytes per donor. These data were then summarised at three levels: (1) at the level of each individual cluster (to allow for detailed analysis of cluster volume); (2) at the level of each individual cell (to allow for detailed analyses of cluster number per cell) and (3) at the level of each individual donor (to provide a single data point for each participant which represented their median CR1 cluster number or mean cluster volume).

Initial exploratory analyses used the data at the level of the individual donor to examine the influence of donor variables on CR1 copy number, CR1 cluster number and cluster volume. The donor variables explored in our analyses were basic demographic data (age and gender), other variables reported to influence CR1 copy number (α+thalassaemia genotype and sickle cell genotype)^40,42,63^, ABO blood group (repeatedly associated with malaria in this population)^59,62^ and Knops genotype (*1a*, *2a* and *2b*). Correlation between continuous variables was analysed by linear regression, and differences between means were analysed by one-way ANOVA or unpaired t tests.

To examine factors potentially influencing CR1 cluster number, the data were then analysed at the level of the individual cells. To permit inclusion of all the data collected and account for the intra-donor individual variation in the number of clusters per cell, a two-level hierarchical mixed effects Poisson regression model was used. Donor identifier and date the experiment was performed were included as random effects. Each donor variable (as above) was then examined in turn as a fixed effect. In view of the well documented relationship between CR1 cluster number and CR1 copy number^25,32,33^, each variable was examined both with and without CR1 copy number in the model.

Finally, to examine factors potentially influencing CR1 cluster volume and account for both intra-individual and intra-cell variation in volume of clusters, a three-level hierarchical mixed effects linear regression model was used with both donor identifier and cell identifier included as random effects. Each donor variable was then examined as a fixed effect with and without CR1 copy number in the model. Where t values were significant, bootstrapping with 1000 iterations was performed to provide an equivalent p value for ease of interpretation.

Analyses were performed using R statistical software (R Foundation for Statistical Computing, Vienna, Austria)^66^ using the packages “plyr”, “ggplot2”, “boot” and “lme4”^67–69^. Graphs were drawn using GraphPad Prism 7 (GraphPad Software, Inc., La Jolla, CA, USA).

### Data availability

Data at the level of individual donors are in Supplementary dataset 1, data at the level of individual cells are in Supplementary dataset 2, and data at the level of individual clusters are in Supplementary dataset 3.

## Electronic supplementary material

Supplementary Information

Dataset 1

Dataset 2

Dataset 3
