# Supplementary material for: No Evidence that Knops Blood Group Polymorphisms Affect Complement Receptor 1 Clustering on Erythrocytes
Source: Sci Rep. 2017 Dec 19;7:17825. doi: 10.1038/s41598-017-17664-9 (PMC5736761; doi:10.1038/s41598-017-17664-9)
Supplement: Supplementary file 1 — Supplementary Information [file 41598_2017_17664_MOESM1_ESM.pdf]

No Evidence that Knops Blood Group Polymorphisms Affect Complement Receptor 1 Clustering on Erythrocytes

Swann OV, Harrison EM, Opi DH, Nyatichi E, Macharia A, Uyoga S, Williams TN, Rowe JA

Supplementary Information:

Supplementary Text S1 - description of Supplementary Videos 1 and 2

Supplementary Figures 1-8

Supplementary Text S1 – description of Supplementary Videos 1 and 2

Supplementary Videos 1 and 2

These videos animate a series of XY images of a single erythrocyte, which in turn make up a Z-stack. The bottom right quadrant shows the current XY image and the top left shows where the image occurs as the video moves through the Z-stack. Clusters can be seen to start, grow and recede as the animation moves through the steps in turn.

Video 1: the fluorescent CR1 clusters on the single erythrocyte are represented in white.

Video 2: the same images are shown, but with the addition of the cluster identification protocol, each separate cluster is identified by a unique colour.

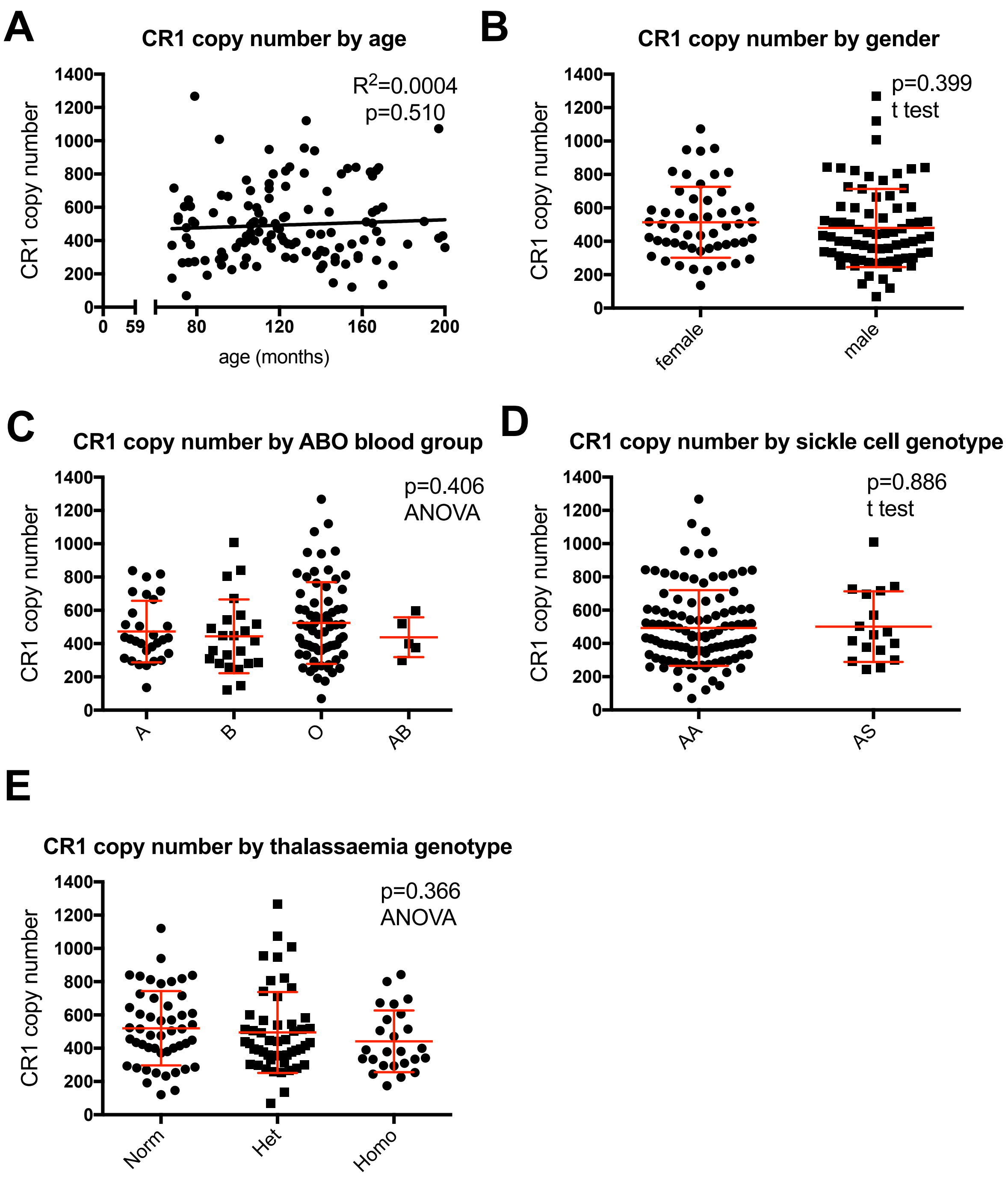

Figure S1. Preliminary exploration of the association of donor variables and erythrocyte CR1 copy number (mean number of CR1 molecules per cell). A) host age B) gender C) ABO blood group D) sickle cell genotype E) α+thalassaemia genotype (Norm, normal α globin; Het, heterozygote; Homo, homozygote). Red bars represent mean and standard deviation. Correlation was analysed by linear regression, and differences between means were analysed by one-way ANOVA or t test as shown above.

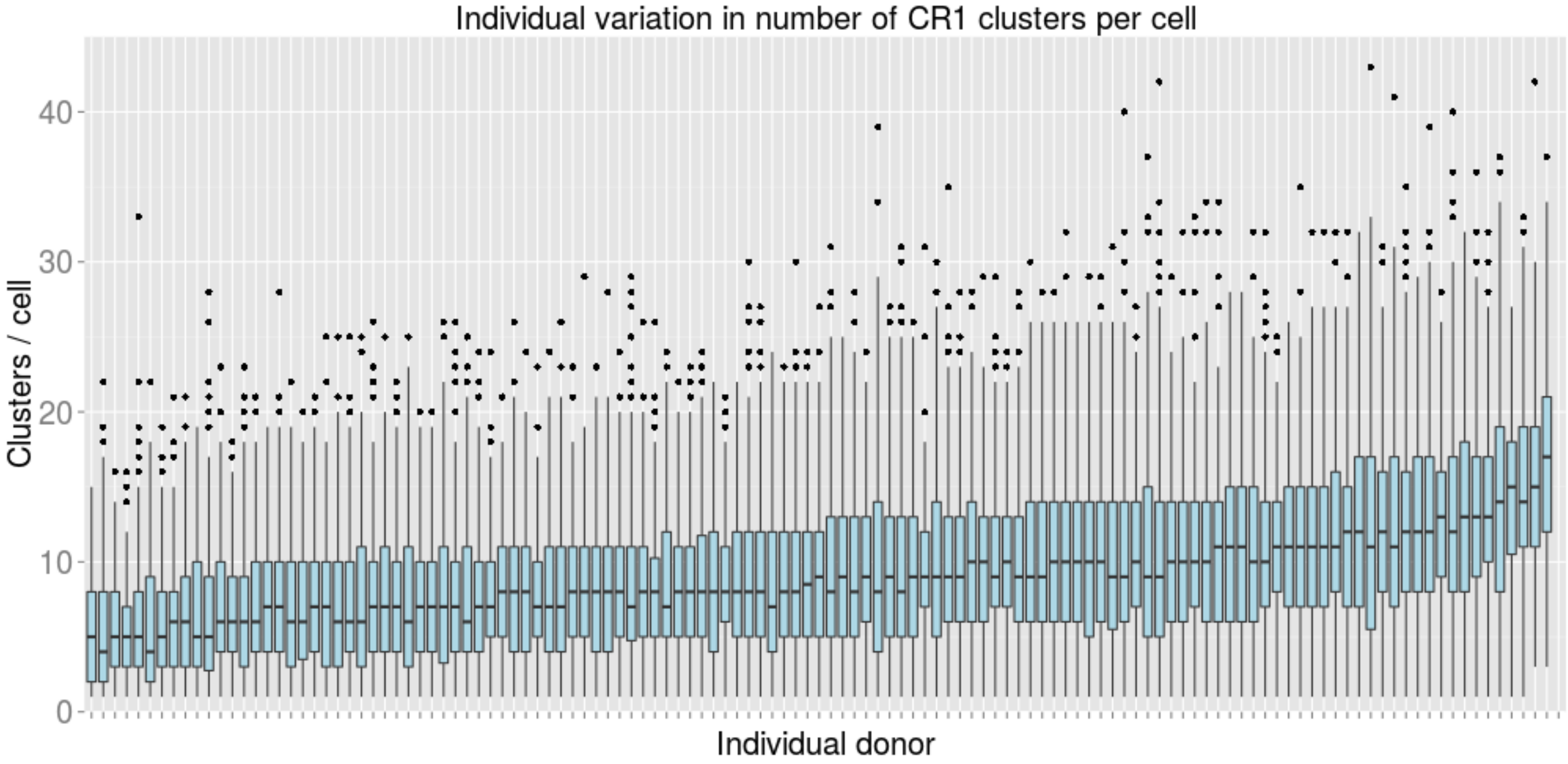

Figure S2. Box plot to illustrate within individual variation in number of CR1 clusters per cell. Line = median, box = IQR, whiskers = data within 1.5 *IQR of lower/upper quartiles. Each box represents an individual donor, ordered by increasing CR1 copy number.

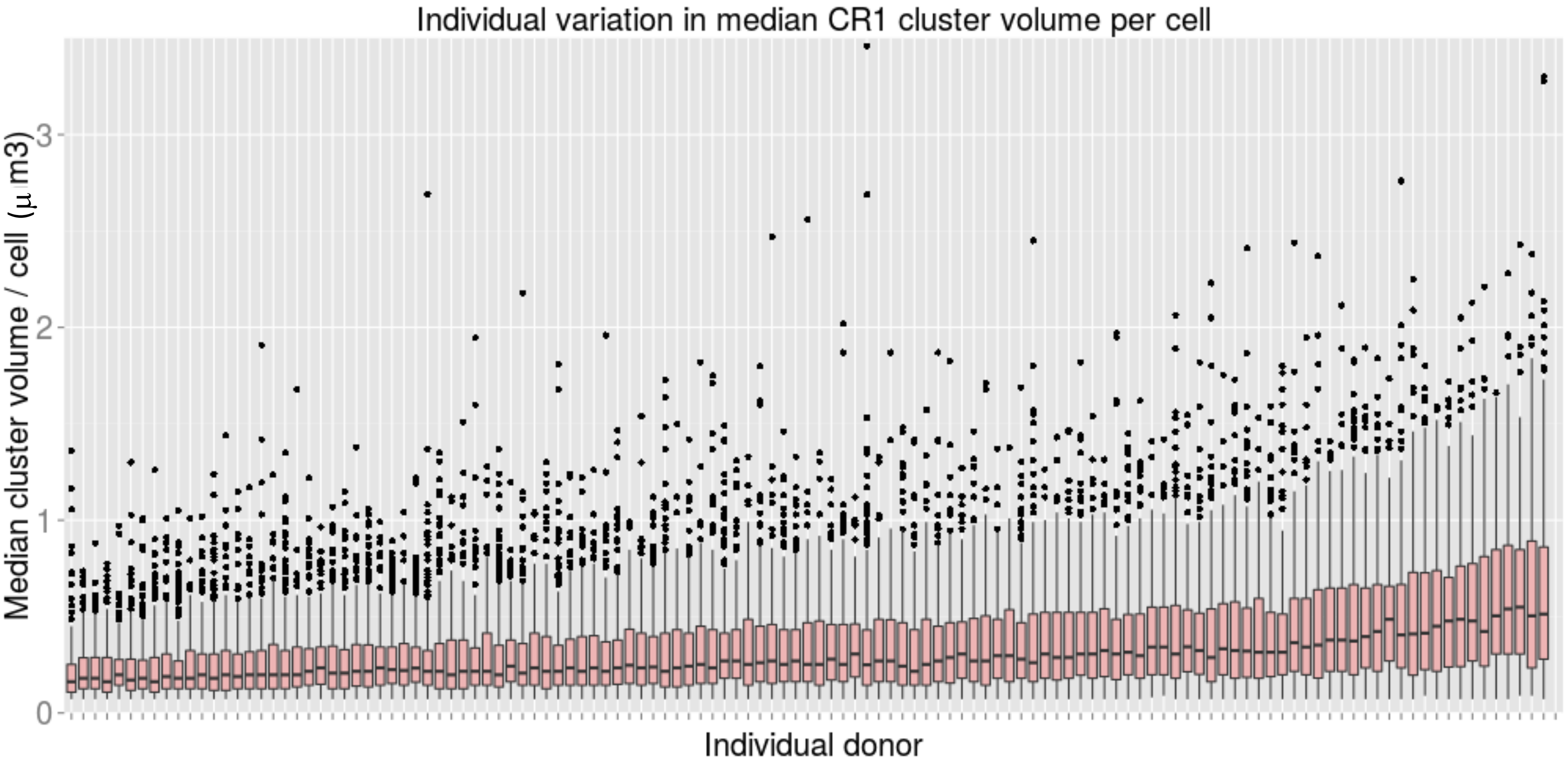

Figure S3. Box plot to illustrate within individual variation in median cluster volume per cell. Line = median, box = IQR, whiskers = data within 1.5 *IQR of lower/upper quartiles. Each box represents an individual donor, ordered by increasing CR1 copy number

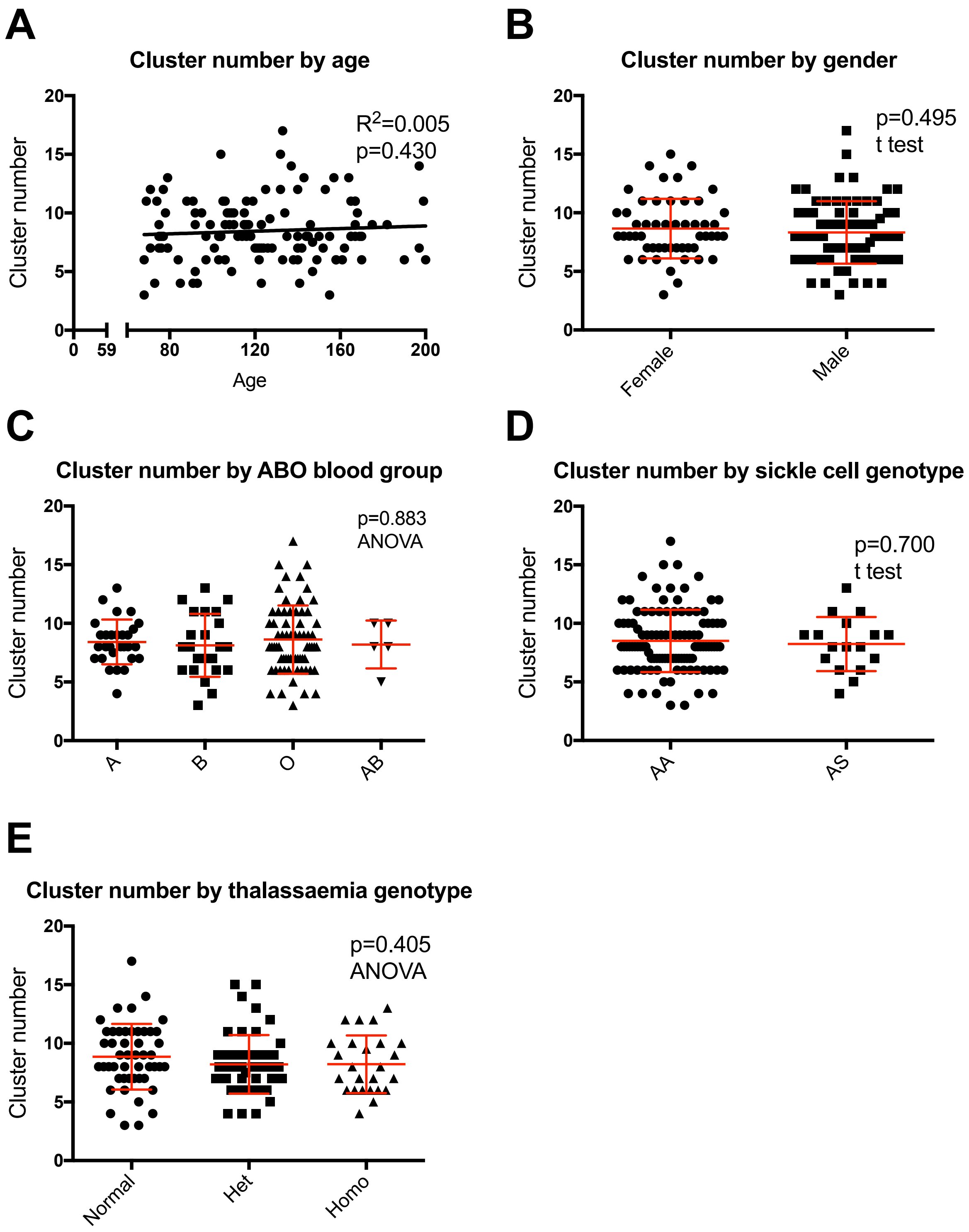

Figure S4. Preliminary exploration of the association of donor variables and median erythrocyte cluster number. Red bars represent mean and standard deviation. A) host age B) gender C) ABO blood group D) sickle cell genotype E) α+thalassaemia genotype (Normal, normal α globin; Het, heterozygote; Homo, homozygote). Red bars represent mean and standard deviation. Correlation was analysed by linear regression, and differences between means were analysed by one-way ANOVA or t test as shown above.

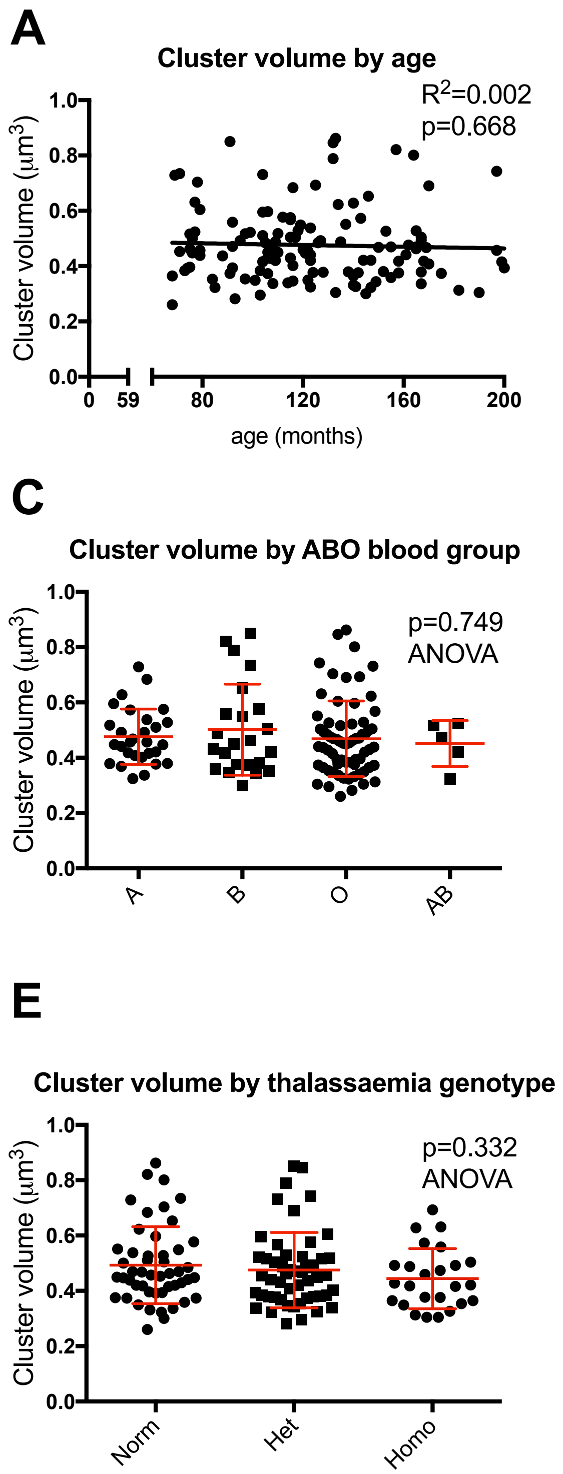

Figure S5. Preliminary exploration of the association of donor variables and mean erythrocyte cluster volume. Red bars represent mean and standard deviation. A) host age B) gender C) ABO blood group D) sickle cell genotype E) α+thalassaemia genotype (Norm, normal α globin; Het, heterozygote; Homo, homozygote). Red bars represent mean and standard deviation. Correlation was analysed by linear regression, and differences between means were analysed by one-way ANOVA or t test as shown above.

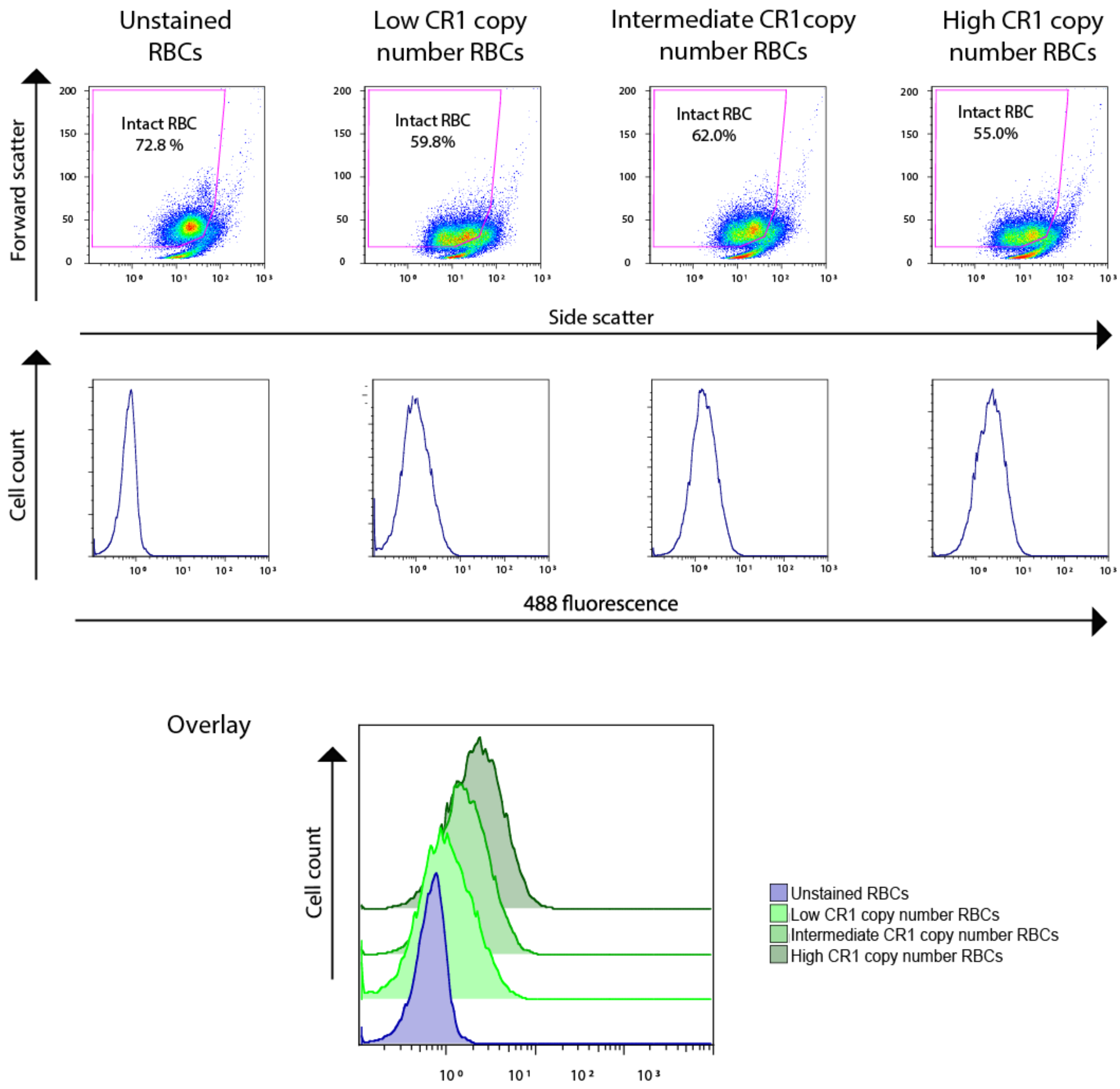

Figure S6. Immunofluorescence staining and flow cytometry to determine erythrocyte CR1 copy number. Representative flow cytometry analyses comparing unstained red blood cells (RBCs) with RBCs from individuals with low, intermediate and high CR1 copy numbers. Dot plots (top row) show forward scatter against side scatter with a gating strategy to include only intact cells. Histograms (middle row) show Alexa Fluor^{488} fluorescence from J3D3 (α-CR1) antibody staining for all intact RBCs in each sample. Increasing fluorescence is seen with higher CR1 copy number. The overlay (bottom row) displays the comparative Alexa Fluor^{488} fluorescence of intact RBCs from the four donors.

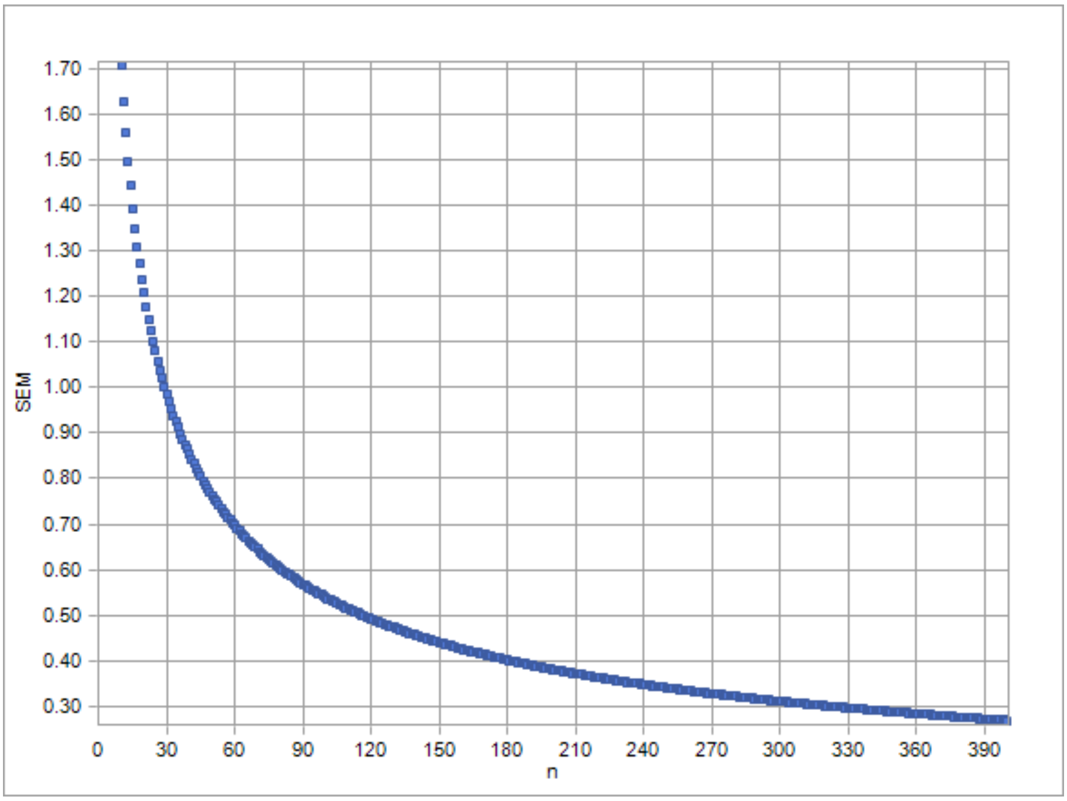

Figure S7. Determining the optimal number of erythrocytes to image. The plot illustrates how the standard error of the mean (SEM) of the number of CR1 clusters per erythrocyte alters with increasing number of erythrocytes imaged (n) from a single donor. Based on these data, it was decided to image 200 erythrocytes for each sample, as reductions in SEM beyond this point were small.

A

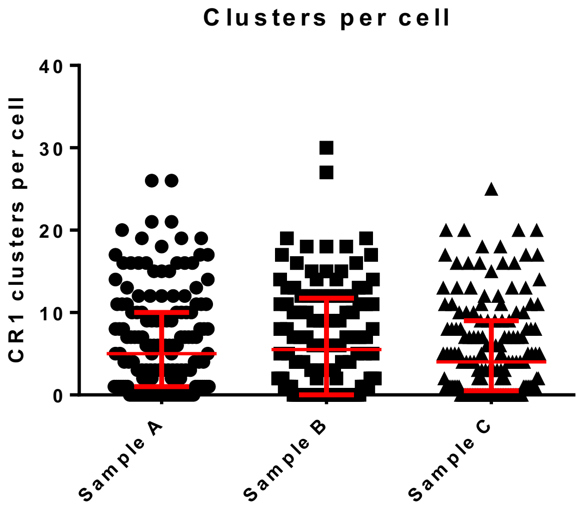

B

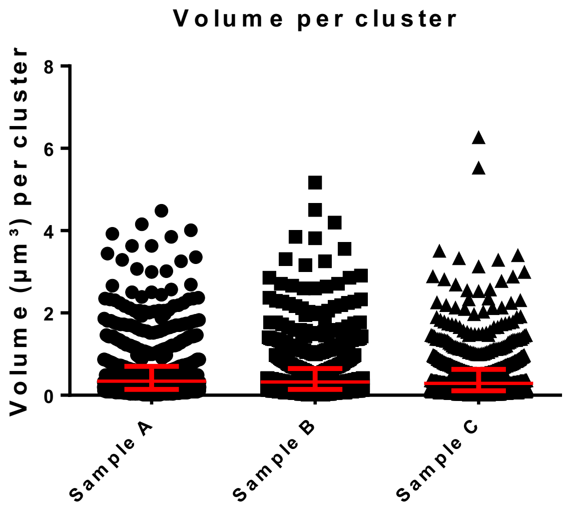

Figure S8. CR1 cluster number and volume for three samples taken over consecutive days from one donor. A) CR1 cluster number per cell. Each data point represents one of the 200 cells imaged. The median and interquartile range are shown as red bars. There were no significant differences between the sample medians (Kruskal-Wallis test p=0.337). B) Volume per CR1 cluster. The median and interquartile range are shown as red bars. The difference between the median cluster volumes was of borderline significance (Kruskal-Wallis test p= 0.054), but the absolute difference between the medians was extremely small.
